# Loss of Mediator complex subunit 13 (MED13) promotes resistance to alkylation through cyclin D1 upregulation

**DOI:** 10.1093/nar/gkaa1289

**Published:** 2021-01-14

**Authors:** Miłosz Roliński, Nicola Pietro Montaldo, Merdane Ezgi Aksu, Sarah L Fordyce Martin, Alessandro Brambilla, Nicolas Kunath, Jostein Johansen, Sten Even Erlandsen, Nina-Beate Liabbak, Kristin Rian, Magnar Bjørås, Pål Sætrom, Barbara van Loon

**Affiliations:** Department of Clinical and Molecular Medicine, Norwegian University of Science and Technology, 7049 Trondheim, Norway; Department of Clinical and Molecular Medicine, Norwegian University of Science and Technology, 7049 Trondheim, Norway; Department of Clinical and Molecular Medicine, Norwegian University of Science and Technology, 7049 Trondheim, Norway; Department of Clinical and Molecular Medicine, Norwegian University of Science and Technology, 7049 Trondheim, Norway; Department of Clinical and Molecular Medicine, Norwegian University of Science and Technology, 7049 Trondheim, Norway; Department of Clinical and Molecular Medicine, Norwegian University of Science and Technology, 7049 Trondheim, Norway; Bioinformatics core facility - BioCore; Norwegian University of Science and Technology (NTNU), 7491 Trondheim, Norway; Genomics core facility, Norwegian University of Science and Technology (NTNU), 7491 Trondheim, Norway; Department of Clinical and Molecular Medicine, Norwegian University of Science and Technology, 7049 Trondheim, Norway; Department of Clinical and Molecular Medicine, Norwegian University of Science and Technology, 7049 Trondheim, Norway; Department of Clinical and Molecular Medicine, Norwegian University of Science and Technology, 7049 Trondheim, Norway; Department of Microbiology, Oslo University Hospital, 0027 Oslo, Norway; Department of Medical Biochemistry, Oslo University Hospital and University of Oslo, 0372 Oslo, Norway; Department of Clinical and Molecular Medicine, Norwegian University of Science and Technology, 7049 Trondheim, Norway; Bioinformatics core facility - BioCore; Norwegian University of Science and Technology (NTNU), 7491 Trondheim, Norway; K.G. Jebsen Center for Genetic Epidemiology, Norwegian University of Science and Technology (NTNU), 7491 Trondheim, Norway; Department of Computer Science, Faculty of Information Technology and Electrical Engineering, Norwegian University of Science and Technology (NTNU), 7491 Trondheim, Norway; Department of Clinical and Molecular Medicine, Norwegian University of Science and Technology, 7049 Trondheim, Norway

## Abstract

Alkylating drugs are among the most often used chemotherapeutics. While cancer cells frequently develop resistance to alkylation treatments, detailed understanding of mechanisms that lead to the resistance is limited. Here, by using genome-wide CRISPR–Cas9 based screen, we identify transcriptional Mediator complex subunit 13 (MED13) as a novel modulator of alkylation response. The alkylation exposure causes significant MED13 downregulation, while complete loss of MED13 results in reduced apoptosis and resistance to alkylating agents. Transcriptome analysis identified *cyclin D1 (CCND1)* as one of the highly overexpressed genes in MED13 knock-out (KO) cells, characterized by shorter G1 phase. MED13 is able to bind to *CCND1* regulatory elements thus influencing the expression. The resistance of MED13 KO cells is directly dependent on the cyclin D1 overexpression, and its down-regulation is sufficient to re-sensitize the cells to alkylating agents. We further demonstrate the therapeutic potential of MED13-mediated response, by applying combinatory treatment with CDK8/19 inhibitor Senexin A. Importantly, the treatment with Senexin A stabilizes MED13, and in combination with alkylating agents significantly reduces viability of cancer cells. In summary, our findings identify novel alkylation stress response mechanism dependent on MED13 and cyclin D1 that can serve as basis for development of innovative therapeutic strategies.

## Graphical Abstract

**Graphical Abstract:**
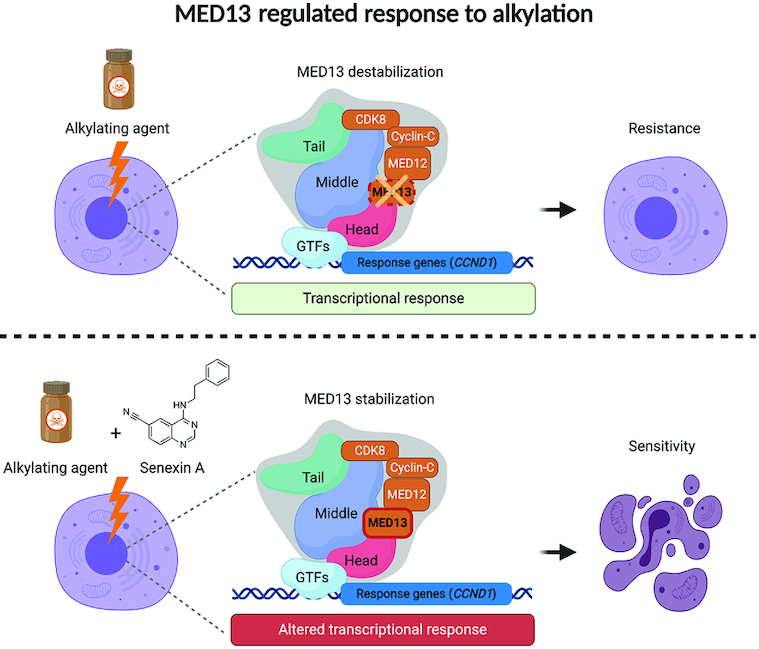
Model of MED13 regulated response to alkylation. Created with BioRender.com.

## INTRODUCTION

Exposure to exogenous and endogenous alkylating agents results in damage of fundamental biomolecules including DNA (1). Approximately 20 000 endogenous DNA lesions are generated in each cell of our body per day (2). Alkylation induced DNA damage can be a source of genome instability, and as such contribute to cancer development (3). Importantly, the harmful properties of alkylating agents can be utilized in clinics to kill fast proliferating cells and treat cancer (4). Though alkylating drugs, like temozolomide (TMZ) are often used in therapy, cancer cells frequently develop resistance to these drugs (5,6). While many factors that facilitate repair of alkylation damage have been identified, key processes contributing to the alkylation resistance remain largely elusive.

Genomic phenotyping and interaction mapping in yeast identified transcription as one of the several novel pathways for alkylation resistance (7,8). We very recently showed that transcription inhibition impairs repair and promotes accumulation of alkylated bases in the genome (9). In addition, several transcription modulators have been suggested to influence response to DNA damaging agents, including Mediator complex (10). Mediator is a large multi-protein complex organized in head, middle and tail, joined with a kinase module. The kinase module is composed of Mediator subunit 13 (MED13), MED12, cyclin C and cyclin-depended kinase 8 (CDK8) (11). The main role of the Mediator is to transduce signals from general transcription factors to RNA polymerase (pol) II. To date, CDK8 was suggested to regulate transcription both positively and negatively (12,13). Several Mediator subunits are known to directly affect gene expression through binding to enhancers and promoters (14). Not surprisingly, several Mediator subunits were shown to be mutated in different cancers (15–17). While transcriptional components, as Mediator, were suggested to influence response to alkylating drugs, their exact importance in drug resistance and cancer therapy is not fully understood.

Besides transcription, cell cycle status was indicated to have important impact on the survival upon exposure to DNA damaging agents. Alterations in the major cell cycle regulators, such as *CCND1* and consequent cyclin D1 overexpression, were associated with both resistance to DNA damaging agents, and genome instability (18). The *CCND1* amplification is one of the major events observed in numerous human cancers (19). Cyclin D1 through activation of CDK4 and subsequent retinoblastoma protein (Rb) hyperphosphorylation, governs transition from G1 to S phase (20,21). The overexpression of cyclin D1 was accordingly demonstrated to result in shorter G1 phase (22,23). Further, cyclin D1 was shown to have unconventional roles through direct impact on DNA repair, as well as in transcriptional control of genes important for chromosomal segregation (24–27). Its expression is regulated both at transcriptional and posttranscriptional level, and several cyclin D1 activators and repressors were identified (20,21). Importantly, depending on the severity of DNA damaging conditions, cyclin D1 levels were reported to be differently regulated (18). How different types of DNA damage influence cyclin D1 status and what are the additional layers of cyclin D1 regulation is currently still unclear.

In this work by performing genome-wide CRISPR–Cas9 based screen we identify MED13 as the novel modulator of response to alkylation exposure. MED13 knock-out (KO) promotes resistance to alkylating agents: methyl methanesulfonate (MMS), TMZ and 1,3-bis[2-chloroethyl]-1-nitrosourea (BCNU, aka Carmustine). The resistance to alkylation is accompanied by reduced apoptosis of MED13 KO cells. Accordingly, upon the exposure wild-type (WT) cells downregulate MED13 to survive. Comparison of MED13 WT and KO transcriptomes identified *CCND1* as one of the most highly overexpressed genes in cells lacking MED13. Additionally, we show that MED13 binds the promoter and enhancer regions of *CCND1* and thus has ability to directly influence its expression. In line with cyclin D1 overexpression we observe that MED13 KO cells have shorter G1 phase. Notably, the observed resistance of MED13 KO cells is directly dependent on cyclin D1 overexpression, and its downregulation counteracts resistance to MMS. The importance of MED13-mediated alkylation response is strongly demonstrated through combinatory treatment with Senexin A, the inhibitor of CDK8/19. Treatment with Senexin A efficiently stabilizes MED13 and in combination with alkylating agents significantly sensitizes cancer cells to alkylation. Taken together, the results of this study suggest a key role of MED13, the subunit of transcriptional Mediator complex, in modulation of resistance to alkylation therapies. This MED13 property has a potential to serve as basis for the design of innovative strategies for cancer therapies.

## MATERIALS AND METHODS

### Cells and cell culture

Near-haploid HAP1 cell line, derived from the KBM-7 cell line, was obtained from Horizon Genomics, Cambridge, UK. Human embryonic kidney 293T (HEK293T), glioblastoma T98G, cervical adenocarcinoma HeLa (CCL-2) and osteosarcoma U2OS cells were obtained from ATCC (USA). Glioblastoma-derived neural stem cells G144 (kind gift from Deo Prakash Pandey, originally established in (28)). All cell lines were cultured under 5% CO_2_ and 37°C. HAP1 cells were cultured in Iscove's modified Dulbecco's medium (IMDM, 12440053, Gibco), supplemented with 10% fetal bovine serum (FBS) (16000044, Gibco), 1% Penicillin–Streptomycin (15140122, Gibco) and 1% l-glutamine (25030081, Gibco). T98G cells were cultured in minimum essential Eagle's medium (M5650, Sigma-Aldrich) supplemented with 10% FBS, 1% Penicillin–Streptomycin, 1% l-glutamine, 1% sodium pyruvate (S8636, Sigma-Aldrich), 1% MEM nonessential amino acids (M7145, Sigma-Aldrich). HEK293T, HeLa and U2OS cells were cultured in Dulbecco’s modified Eagle’s medium (D5796, Sigma-Aldrich) supplemented with 10% FBS and 1% Penicillin–Streptomycin, with addition of 1% l-glutamine to HeLa and U2OS cultures. G144 cells were grown on PDL and Laminin coated standard six-well plates. The plates were coated with 5 μg/ml PDL (A003E, Merck Millipore) overnight and 5 μg/ml Laminin (3446-005-01, R&D Systems) for 2 h. G144 cells were maintained in GBM medium [50% Dulbecco′s modified Eagle′s medium nutrient mixture F-12 (11330057, GIBCO) and 50% neurobasal medium (21103-049, GIBCO)] that was supplemented with 1% Penicillin–Streptomycin, 1% MEM nonessential amino acids (M7145, Sigma-Aldrich), 1× Glutamax supplement (35050061, GIBCO), 1× N2 supplement (17502001 ThermoFisher), 1X B-27™ supplement without vitamin A (12507010, GIBCO), 50 μM 2-mercaptoethanol (31350010, GIBCO), 50 μg/ml bovine serum albumin (BSA) (05470, Sigma), 4 μg/ml heparin (H3149-10KU, Sigma), 20 ng/ml epidermal growth factor (EGF) (236-EG-200 R&D Systems), 20 ng/ml fibroblast growth factor (FGF) (100-18B-1MG, Peprotech).

### Lentivirus generation

HEK293T cells were seeded at 40% confluency the day before transfection. Transfection was performed using VSV.G (14888, Addgene) and psPAX2 (12260, Addgene) plasmids, pooled human CRISPR Knockout (GeCKO v2) libraries A and B (1000000048, Addgene) (29), and Lipofectamine 2000 (11668027, Invitrogen) according to the manufacturer's protocol. Fifty-nine hours after transfection, the media was collected and virus ultracentrifuged at 125 682 × g for 2 h at 4°C. The lentiviral pellet was resuspended in DMEM medium supplemented with 1% BSA (15561020, Invitrogen), aliquoted and stored at −80°C.

### Genome-wide CRISPR–Cas9 screen

2 × 10^6^/well HAP1 cells were seeded in a six-well plate in the presence of 8 μg/ml polybrene (107689, Sigma-Aldrich), and lentivirus carrying GeCKOv2 library A and B added, to achieve multiplicity of infection (MOI) 0.4 and incubated at 37°C overnight. The total number of transduced cells was calculated to achieve at least 300× coverage per each single guide (sg) RNA construct. Next day, cells from each well were transferred to T175 flasks and cultured in IMDM medium supplemented with 0.75 μg/ml puromycin (InvivoGen). Puromycin selection was performed continuously for 7 days. Upon selection 5 × 10^7^ cells were collected as the pre-treatment control, while the rest was transferred to new T175 flasks in three technical replicates and incubated overnight to reach 50% confluency on the day of treatment. Cells were treated for 3 or 7 days with 125 μM MMS (129925, Sigma-Aldrich), or IMDM medium alone in the case of the control. Next, 5 × 10^7^ cells were collected from each condition, represented in triplicate and genomic DNA isolated using DNA Isolation Kit for Cells and Tissues (11814770001, Roche). Illumina libraries were prepared using two step PCR method with Herculase II fusion DNA polymerase (600679, Agilent). The primer sequences are listed in Supplementary Table S1. For the first PCR, 53 reactions were prepared, each containing 2.5 μg gDNA in a final volume of 100 μl using the following: 1 cycle 120 s at 95°C (initial denaturation); 25 cycles, each cycle 20 s at 95°C (denaturation step), 20 s at 67°C (annealing step), 30 s at 72°C (extension step); and 1 cycle 180 s at 72°C (final extension). In the second PCR, each reaction contained 5 μl of pooled amplicons from the first PCR in a final volume of 100 μl and subjected to: 1 cycle 120 s at 95°C; 10 cycles, each cycle 20 s at 95°C, 20 s at 67°C, 30 s at 72°C; and 1 cycle 180 s at 72°C. A total of 11 reactions were prepared per treatment condition. Samples were purified using Agencourt AMPure XP beads (A63880, Beckman) and analyzed on the Caliper LabChip GX Nucleic Acid Analyzer (PerkinElmer). Sequencing libraries were next denatured, diluted and pooled according to the standard Illumina protocols. Pooled libraries were sequenced on a NextSeq 500 Flow Cell High-Output using 1 × 75 bp chemistry, with 2.7 nM library and 20% spiked-in PhiX, run for 81 cycles on read 1 and 9 cycles on read 2 (indexing read). FASTQ files were created with bcl2fastq 2.20.0.422 (Illumina, CA, USA).

### Bioinformatic analysis of the CRISPR–Cas9 screen sequencing results

To define genes as hits based on shRNA depletion data an RNAi gene enrichment ranking (RIGER) score tool was used (30). Java implementation of RIGER (rigerj) was run using both the default Kolmogorov–Smirnov algorithm and with Second Best Rank, using number of random scores to computer per gene set size of 1 000 000. Quality control was performed using MAGeCK-VISPR (31). Plots were generated using custom scripts generated in R v3.4.1 using ggplot2 v3.2.1.

### Generation of HAP1 and G144 MED13 KO cells

SgRNAs targeting *MED13* were designed using the Optimized CRISPR Design tool (http://tools.genome-engineering.org). The oligo pairs encoding the sgRNAs (Supplementary Table S1) were annealed and ligated into pSpCas9(BB)-2A-GFP (PX458) (Addgene plasmid 48138; a gift from Feng Zhang) (32). To generate MED13 knock-out cells: (i) HAP1 WT cells were transfected using Viromer RED transfection reagent kit (VR-01LB-00, Lipocalyx) according to the manufacturer protocol. GFP positive cells were selected by fluorescence-activated cell sorting (FACS) and seeded as single clones into a 96-well plate. (ii) G144 cells were transfected with Lipofectamine 2000 (11668019, ThermoFisher Scientific) by using manufacturer protocol and selected with 1 μg/ml puromycin for 48h. Next, single GFP positive cells sorted by FACS in each well of a 96-well plate precoated with PDL and Laminin. The single clones were grown and propagated. Inactivation of *MED13*, and consequent loss of MED13 protein in both HAP1 and G144 candidate clones was confirmed by sequencing (using primers in Supplementary Table S1) and immunoblot analysis, respectively.

### Viability assays

Cell viability was evaluated using the PrestoBlue™ Cell Viability Assay (A13262, Invitrogen). 2 × 10^4^ HAP1, or 1.5 × 10^4^ G144 cells were seeded per well of a 96-well plate and incubated for 24 h. Next, cells were treated with MMS, TMZ, BCNU (concentrations indicated in the figures) or media alone for 72 h. Control wells in TMZ and BCNU viability assay were treated with 0.5% DMSO. At the end of the treatment PrestoBlue™ Cell Viability Reagent was added and after 2 h incubation the absorbance measured at 570 nm with reference wavelength 600 nm. The cell viability was calculated using Eq. (1):

$$\begin{equation*}{\rm Cell}\ {\rm viability}\, (\%) = \left( {\frac{{{\rm ODtest} - {\rm ODblank}}}{{{\rm ODcontrol} - {\rm ODblank}}}} \right)\ \times \ 100\end{equation*}$$

where ODtest is the optical density of cells exposed to damaging agent, ODcontrol is the optical density of the control untreated sample, and ODblank is the optical density of wells with media alone.

### Colony formation assay

300 cells/well were plated in six-well plates. Cells were treated for 72 h with increasing concentrations MMS, TMZ or BCNU (indicated in the Figures). Control wells in TMZ and BCNU experiments included 0.5% DMSO. TMZ and BCNU were diluted in DMSO. Upon treatment cells were allowed to form colonies for 5 days. Cell culture plates were then gently washed with PBS and colonies stained with 0.5% crystal violet solution in 6% glutaraldehyde for 30 min. Excess stain was removed by washing repeatedly with PBS. The colony area percentage was quantified using ColonyArea plugin for ImageJ (33), according to Eq. (2):

$$\begin{eqnarray*}&&{\rm Colony}\ {\rm area}\ \ ( \% )\nonumber\\ && = \ \left( {\frac{{\# {\rm{\ of\ pixels\ in\ the\ region\ with\ an\ intensity\ above\ zero\ }}}}{{{\rm{Total\ }}\# {\rm{\ of\ pixels\ in\ the\ same\ region}}}}} \right)\ \times \ 100\end{eqnarray*}$$

The graphs are presented as normalized colony area percentage calculated using Eq. (3):

$$\begin{eqnarray*}&& {\rm Normalized}\ {\rm colony}\ {\rm area}\ \ \left( \% \right) \nonumber\\ &&= \ \left( {\frac{{{\rm Colony}\ {\rm area}\ \left( \% \right)\ {\rm treated}\ {\rm well}}}{{\left( {\left( {{\rm Colony}\ {\rm area}\ \left( \% \right)\ {\rm untreated}\ {\rm well}\ 1} \right)\left( {{\rm Colony}\ {\rm area}\ \left( \% \right)\ {\rm untreated}\ {\rm well}\ 2} \right)} \right)/2}}} \right)\nonumber\\ && \times \ 100\end{eqnarray*}$$

### RT-qPCR analysis of mRNA levels

HAP1 cells were treated with 125 μM MMS or media alone for 72 h, washed twice with PBS, pelleted, snap frozen and stored at −80°C. For mRNA levels analysis total RNA was isolated using RNeasy mini kit (74106, Qiagen), with inclusion of on-column DNase I digestion (79254, Qiagen), according to the manufactured protocol. Total RNA integrity was evaluated via gel electrophoresis analysis of the rRNA 28–18 s ratio and quantified using NanoDrop^®^ (ND-1000 V3.7.1; Thermo Fisher Scientific). Total RNA was diluted to a concentration of 10 ng/μl and reverse transcribed to complementary DNA (cDNA) using high capacity cDNA reverse transcription kit (4368814, Thermo Fisher): 10 μl of total RNA were mixed with 4 mM dNTPs and 50 units of multiverse reverse transcriptase in a final reaction volume of 20 μl in RT buffer supplemented with random primers. The reaction was incubated for 2h at 37°C and 5 min at 85°C. The resulting cDNA was then diluted 1:10 and 2 μl were used for real time-quantitative polymerase chain reaction (RT-qPCR) using Power SYBR Green master mix (4368708, Applied Biosystems) in a total volume of 10 μl with 0.2 μM primers. qPCR experiments were performed in technical triplicates using StepOnePlus v2.3 real-time PCR system (Applied biosystems) following thermocycling parameters: initial step at 95°C for 10 min, followed by denaturation 95°C for 15 s; annealing and extension for 60°C for 1 min, for a total of 40 cycles. Primers targeting *MED13*, *CCND1*, *RELN* and *GAPDH* transcripts are depicted in Supplementary Table S1. Relative expression levels were calculated using the 2ΔΔCt method for qPCR analysis by normalizing to housekeeping gene *GAPDH*.

### Whole cell extract preparation and immunoblot analysis

Cells were treated with 125 μM MMS, or media alone in case of negative control, for 72 h. Next, the whole cell extracts (WCE) were prepared by resuspending pellet from 0.5 × 10^6^ cells in 15 μl hypotonic lysis buffer (20 mM HEPES pH 7.9, 2 mM MgCl_2_, 0.2 mM EGTA, 10% glycerol, 0.1 mM PMSF, 2 mM DTT, 1× Protein Inhibitor Cocktail (Roche)) and incubated on ice for 5 min, followed by three freeze-thaw cycles. Cell suspension was supplemented with 140 mM NaCl and 0.5% NP-40 and incubated 20 min on ice. Next, cell pellet was diluted with 50 μl lysis buffer containing 140 mM NaCl and sonicated using Bioruptor (Diagenode) (5 min; 30 s ON per minute). Samples were centrifuged 10 min, 12 000g at 4°C and supernatant representing WCE collected. For the immunoblot analysis, proteins were separated on NuPage 4–12% Bis–Tris polyacrylamide gel (NP0321, Invitrogen), and transferred to 0.45 μm PVDF membrane in transfer buffer (192 mM glycine, 25 mM Tris base, 5% MetOH). The proteins of interest were detected using specific primary antibodies (anti-MED13 (Novus Biologicals, NB100-60642 1:1000), anti-Tubulin (Cell Signaling, 2144, 1:40 000), anti-β-actin (Sigma, A1978, 1:10000), anti-cyclin D1 antibody (Abcam, ab134175, 1:1000), anti-Phospho Ser15 (P-S15) p53 (Cell Signaling, 9284, 1:1000), anti-P-Ser727 STAT1 (Abcam, ab109461, 1:1000)), and corresponding secondary antibodies: polyclonal swine anti-rabbit immunoglobulins/HRP (PO399, Dako Denmark 1:3000) and infrared (IR) Dye-conjugated secondary antibodies (Li-COR Biosciences, 925-32210, 1:15000). The signal was visualized in case of HRP antibodies by SuperSignal West Femto Maximum Sensitivity Substrate (34096, Thermo Scientific) and ChemiDoc imager system (Bio-Rad), and the IR signal by the Odyssey Scanner, LI-COR Biosciences. Protein levels were quantified using ImageJ software.

### RNA sequencing and bioinformatic processing

0.11 × 10^6^ HAP1 WT or HAP1 MED KO clones (cl.) 10 and 17 were seeded per six-well plate. Next day cells were treated with 125 μM MMS, or media alone in case of negative control, for 72 h. Total RNA was purified with RNeasy mini kit (74106, Qiagen), in presence of DNase I (79256, Qiagen), according to the manufacturer's protocol. RNA library preparation, and sequencing of HAP1 samples, were carried out by the BGI Genomics, China. The libraries were screened on the Bioanalyzer (Agilent), pooled in equimolar concentrations, and sequenced using a paired-end (PE), 100 bp stranded mRNA library protocol (B02) on a BGIseq platform. PE FASTQ files were processed to remove adapter sequences and low-quality reads using Trimmomatic v0.33 (34). Reads passing filtering were mapped to the GRCh38.84 reference genome using STAR aligner v2.4.0 (35). Annotation and gene counts were obtained using HTseq v0.6.0 (htseq-count) counting exon features and reporting Ensembl Gene IDs (36). Data normalization and differential expression analysis was performed using limma voom function v3.32.10 (37) in R v3.4.1, filtering out genes with expression (total normalized read counts) less than the total number of samples. The RNA sequencing data reported in this paper are available in GEO under accession GSE147366.

### Chromatin immunoprecipitation

HAP1 WT cells were crosslinked with 1% formaldehyde for 10 min and quenched with 0.11 mM glycine for 5 min. Cells were washed with ice-cold PBS and harvested. Cell pellets were resuspended in cell lysis buffer (100 mM Tris–HCl pH 8, 10 mM DTT) and incubated 15 min on ice, and 15 min at 30°C. Next, nuclei were pelleted and washed with buffer A (10 mM EDTA pH 8, 10 mM EGTA, 10 mM HEPES pH 8, 0.25% Triton X-100), followed by buffer B (10 mM EDTA pH 8, 0.5 mM EGTA, 10 mM HEPES pH 8, 200 mM NaCl). Nuclei were lysed in lysis buffer (50 mM Tris–HCl pH 8, 10 mM EDTA and 1% SDS) and chromatin sheared to 200–250 bp DNA fragments by sonication with Bioruptor (Diagenode) for 30 min; 30 s ON per minute. 25 μg of chromatin was next precleared for 2 h at 4°C, and incubated with 2 μg antibody (anti-MED13 (Novus Biologicals, NB100-60642) or rabbit IgG (Diagenode, C15410206) in ChIP buffer (16.7 mM Tris–HCl pH 8, 167 mM NaCl, 1.2 mM EDTA, 0.01% SDS and 1.1% Triton X-100) overnight at 4°C. The DNA–protein–antibody complexes were isolated using A dynabeads (88802, Thermo Scientific) and washed using sequentially: low salt wash buffer (16.7 mM Tris–HCl pH 8, 167 mM NaCl, 0.1% SDS, 1% Triton X), high salt wash buffer (16.7 mM Tris–HCl pH 8, 500 mM NaCl 0.1% SDS, 1% Triton X) and LiCl wash buffer (250 mM LiCl, 0.5% NP40, 0.5% Na-deoxycholate, 1 mM EDTA, 10 mM Tris–HCl pH 8). Proteinase K treatment was performed for 1 h at 50°C with 10 mM EDTA, 40 mM Tris–HCl pH 6.5 and 20 μg proteinase K (AM2548, Invitrogen). The DNA was purified with phenol-chloroform, ethanol precipitated and analyzed by qPCR. The qPCR experiments were performed in technical triplicates using following thermocycling parameters: 95°C for 10 min, followed by 40 cycles 95°C for 15 s; 60°C for 1 min, in StepOnePlus v2.3 Real-Time PCR System (Applied Biosystems). Levels of immunoprecipitated DNA is expressed as relative occupancy = (% input of tested condition)/(% input of control condition). The ‘% input’ value represents the enrichment of MED13 on specific region of the genome. Primer sequences are listed in the Supplementary Table S1.

### Cell cycle analysis

1 × 10^6^ HAP1 WT and MED13 KO cl.10 cells were seeded in T25 flasks and next day treated with 125 μM MMS or IMDM medium alone for 72 h. After treatment, cells were collected, washed once with 1 ml cold PBS, fixed in 1 ml of ice-cold 100% methanol and centrifuged at 200g at 4°C. Cell pellets were washed once with PBS and treated with 0.1 mg/ml RNase (R5503; Sigma-Aldrich) at 37°C for 30 min in 200 μl. Next, 200 μl of 50 μg/ml propidium iodide (PI) solution (P4864; Sigma-Aldrich) was added and samples incubated for 30 min at 37°C, followed by FACS analysis. Cells were analyzed using FlowJO 10.6.1. Forward scatter FSC-A and side scatter SSC-A were used to identify cell population; PI fluorescence pulse area (PI-A) and PI fluorescence pulse width (PI-W) were used to identify single cells. Cell cycle phases were analyzed in PI histogram plot. Data is available in FlowRepository under accession FR-FCM-Z32R.

### Immunofluorescence analysis of γH2AX

The six-well plates were coated with 5 μg/ml poly-d-lysine (p0899, Sigma-Aldrich) and 0.35 × 10^6^ HAP1 WT or MED13 KO cl.10, cl.17 cells seeded per well. Next day, cells were treated with 125 μM MMS for 72 h. After the treatment, cells were washed two times with PBS and fixed with 4% paraformaldehyde (104005.1000, Millipore) for 15 min. Cells were washed three times with PBS and permeabilized with 0.1% Triton X-100 in PBS for 40 min at RT. The blocking was performed with 0.1% Triton X-100, 5% BSA and 5% goat serum (10000C, Life Technologies) in PBS for 40 min at RT. Cells were incubated 2 h at RT with primary antibody Anti – pSer139 H2A.X clone JBW301 (05-636, Millipore, 1:80) diluted in 0.1% Tween-20, 0.5% BSA and 0.5% goat serum. Cells were washed with 0.1% Triton X-100 in PBS and incubated 1 h at RT with secondary antibody Alexa Fluor 488 goat anti-mouse IgG1 (A21121, Invitrogen, 1:500) diluted in 0.1% Triton X-100 in PBS. Cell were washed three times with 0.1% Triton X-100 in PBS, stained and mounted with Prolong Gold Antifade reagent with DAPI (P36935, Invitrogen). The samples were imaged using a Zeiss LSM880 confocal microscope (Zeiss, Jena, Germany) at a 40x magnification (1.4 NA). Quadratic images with a side length of 354.25 μm were taken in 24+/–2 *z*-planes with a *z*-plane-interval of 0.5 μm (*x*- and *y*-length of a pixel equaling 0.346 μm). Images were taken at three random places per slide (one slide equal one statistical unit/condition) by an experimenter not acquainted with the study design. After three-dimensional rendering using the software Imaris 8.2 (Bitplane, Zurich, Switzerland), nuclei were identified by modelling spherical objects (‘spots’) with a diameter of 10 μm around all DAPI-positive areas. DAPI areas >50% outside the imaging area were not considered for counting. The spots were used to define the intranuclear area by ‘masking’ the channel containing the γH2AX/Alexa488 fluorescent signal, setting the voxels outside the spots to a fluorescent signal equaling zero. Within the masked channel, high-intensity spots were identified by modelling spherical objects with a 1 μm diameter. Two random images with a relevant number of double-strand break spots were used as a reference measurement, in which spots were first identified using the automatic spot detection method provided by Imaris (‘quality’ filter, Gaussian filter with background subtraction), with an automatic thresholding algorithm (38). Next, the threshold defined in the two initial images was taken as a reference for ‘spots of interest’. All subsequent γH2AX spots were detected using the same threshold criterion after background subtraction to avoid any arbitrary judgement of what spot qualifies as relevant. The number of γH2AX spots was normalized to the number of DAPI nuclei in the image area.

### Flow cytometric determination of apoptosis by annexin V/propidium iodide double staining

Twenty-four hours after seeding HAP1 cells were treated with 125 μM MMS for 72 h. Treated and untreated cells were the harvested, washed with cold PBS and diluted to 1 × 10^6^ cells/ml in 1× annexin-binding buffer. 100 μl of cell suspension were then labeled with Annexin V Alexa Fluor^®^ 488 and propidium iodide (PI) (Sigma) according to the manufacturer's protocol, incubated for 15 min at room temperature and analyzed on a BD FACS Aria II (BD Biosciences). The fraction of apoptotic cells was determined by using the FlowJo, LLC software (USA). Unstained and single stained cells were used as controls. PI negative and Annexin V positive cells we considered apoptotic, and cells that are positive to both PI and Annexin V considered necrotic. Data is available in FlowRepository under accession FR-FCM-Z32T.

### siRNA mediated knock-down of cyclin D1

HAP1 WT and MED13 KO cells were seeded to reach 80% of confluency on the day of transfection. Cells were transfected using 7.5 pmol of siRNA and 7.5 μl Lipofectamine 3000 transfection reagent (L3000008, Invitrogen). The sequences of scrambled and *CCND1 (cyclin D1)* targeting siRNA are listed in Supplementary Table S1. Forty-eight hours post-transfection, cells were treated with different MMS concentrations as indicated in the figures and viability determined using the viability assay.

### Overexpression of cyclin D1

HAP1cells were transfected with pcDNA3-Myc-cyclinD1(WT) (Addgene, 122300) or pEGFP-C1 (Clontech) as mock and transfection control using Lipofectamine 3000 transfection reagent. Twenty-four hours post-transfection, cells were seeded into 96-well plate for viability assay, and a T75 flask for RT-qPCR and immunoblot analysis to confirm overexpression. Next, cells were treated with MMS (concentrations indicated in the figures) or media alone for 72 h for viability assay.

### Combinatory treatment with Senexin A and MMS

HAP1 WT and MED13 KO cl. 10, HeLa, T98G or U2OS cells were seeded in 96-well plates to reach 15% confluency the next day. Cells were first pretreated with Senexin A (HY-15681, MedChemExpress) or 0.125% DMSO for 2 h, followed by addition of modified media without, or with MMS or TMZ and 70 h incubation; all respective drug amounts are indicated in the Figures. Viability and immunoblot analysis were performed as described above.

### Statistical analysis

The differences in survival upon treatment with alkylating agents were analyzed (Figures 2B–D, 4F, 5C–F and Supplementary Figure S2A–C, E–G, 5E) by linear regression. Linear regression analysis, and associated ANOVA testing, was performed using the drc (v2.6-10) package in R according to the authors' code provided in the Supplementary information S1 from Ritz *et al.* (39). Analysis of remaining data was performed using GraphPad Prism 8.2.1. (GraphPad Software, Inc., La Jolla, CA). Statistical significance was determined by two-way ANOVA with Tukey's multiple comparison test (Figure 2I, K and L, 4A, C, E, Supplementary Figure S3B), two-way ANOVA with Dunnet's multiple comparison test (Supplementary Figure S2H and I), two-way ANOVA with Sidak's multiple comparison test (Figure, 4D, H, Supplementary Figure S5F), one-way ANOVA with Dunnett's multiple comparison test (Figure 2F and G, Supplementary Figure S2K and S3A) and one-way ANOVA with Tukey's multiple comparison test (Figure 5B). All data represent mean values ± SEM. *P ≤ 0.05; **P ≤ 0.01; ***P ≤ 0.001; ****P ≤ 0.0001; ns, not significant.

## RESULTS

### CRISPR–Cas9 screen reveals MED13 as one of the top modulators of alkylation resistance

**Figure 1.**
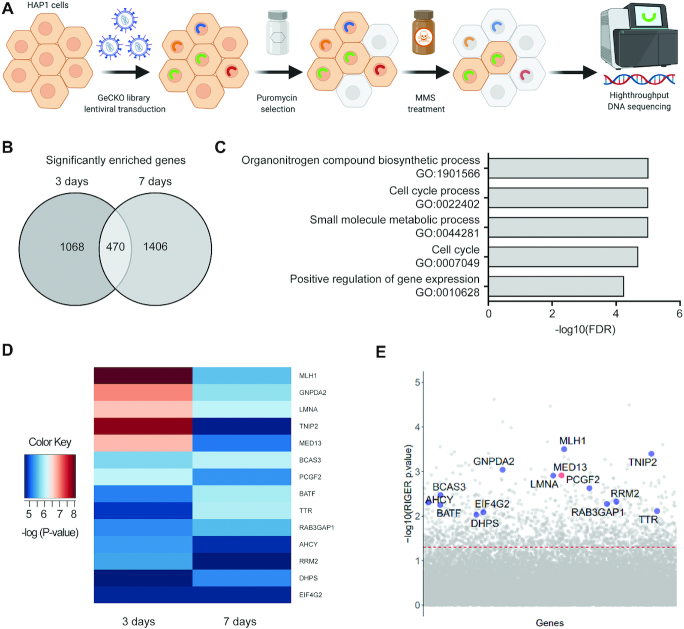
CRISPR–Cas9 screen identified *MED13* subunit of the Mediator complex as a potential regulator of response to alkylation exposure. (**A**) Schematic representation of the CRISPR–Cas9 based screen for identification of key factors that regulate alkylation resistance. Briefly, HAP1 cells were transduced with lentivirus carrying GeCKOv2 library. Positively transduced cells were selected with puromycin, treated with 125 μM methyl methanesulfonate (MMS), and collected 3 and 7 days after the treatment initiation. Genomic DNA from surviving cell population was sequenced using high-throughput platform, leading to identification of candidate genes. Created with BioRender.com. (**B**) Venn diagram representation of the common significantly enriched genes (*P*<0.05) upon MMS exposure, at 3 and 7 day time points, determined by RNAi gene enrichment ranking (RIGER) analysis. (**C**) Top five biological processes (BP) gene ontology (GO) terms as determined by the Gene Set Enrichment Analysis (GSEA) of the common gene candidates determined in (B). (**D**) Heatmap representation of the most enriched candidate genes (*P*< 0.01) clustering in the top five BP depicted in (C). (**E**) Scatterplot representation of the most enriched candidate genes that act as potential modulators of response to alkylation (125 μM MMS, 3 days). The red line indicates the adjusted *P*-value significance threshold.

To determine novel factors that regulate response to alkylation treatment, a CRISPR–Cas9 based screen targeting all protein and micro-RNA coding genes was performed in HAP1 cells (Figure 1A). We used haploid genetic screen, as it represents a powerful tool enabling efficient suppression screening (40,41). The HAP1 cells were treated with alkylating drug MMS and collected 3 and 7 days after the treatment initiation. The most significantly enriched sgRNAs, and corresponding gene targets, were determined by RNA interference gene ranking (RIGER) algorithm. This analysis resulted in identification of 1538 and 1876 significantly (*P* < 0.05) enriched gene targets at 3- and 7-day time points, respectively (Figure 1B). Of those, 470 gene targets were common to both time points, thus representing key genes which when inactivated promote resistance to alkylation. Gene ontology (GO) analysis revealed that common gene targets most significantly segregate to biological processes (BP) implicated in cell cycle, regulation of gene expression, and metabolism (Figure 1C and Supplementary Table S2). To identify the most prominent modulators of alkylation response we next analyzed which of the 470 common genes segregate in one or several of the top five BP GO-terms. This led to identification of 14 most prominent targets (Figure 1D and E, Supplementary Table S3), among which *MLH1*, a previously known regulator of alkylation stress response (42,43), thus supporting the screen validity. In addition to *MLH1*, *MED13* was identified as one of the highest significant gene targets (Figure 1D and E, Supplementary Table S3). Similarly, several other Mediator subunits as *MED16* and *MED21* were among significantly enriched targets at both 3 and 7 days of the treatment (Supplementary Figure S1A and B). Taken together, by employing CRISPR–Cas9 screen we identified several novel factors that have capacity to influence alkylation resistance and, as MED13, have been implicated in essential processes including regulation of transcription and cell cycle.

### Loss of MED13 causes resistance to DNA damaging agents

**Figure 2.**
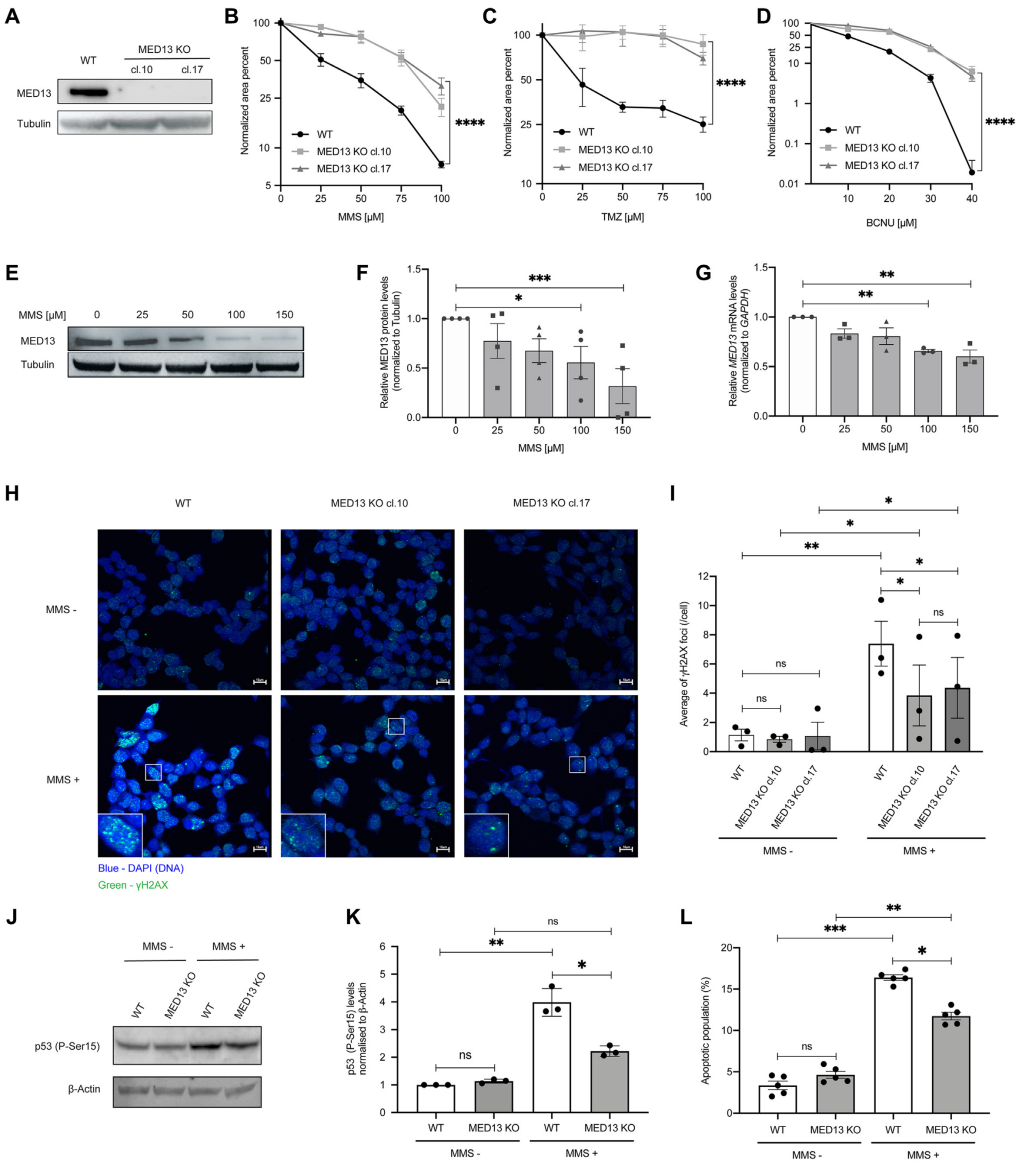
Lack of MED13 causes alkylation resistance. (**A**) Immunoblot analysis of MED13 protein levels in HAP1 wild type (WT) and the two MED13 independent knock-out (KO) clones (cl.10 and cl.17) generated by the CRISPR–Cas9 system. (**B–D**) Colony formation analysis of HAP1 WT and MED13 KO clones upon treatment with indicated amounts of methyl methanesulfonate (MMS) (B) and temozolomide (TMZ) (C) and 1,3-bis[2-chloroethyl]-1-nitrosourea (BCNU) (D). (**E**) Immunoblot analysis of MED13 and Tubulin protein levels upon exposure to the indicated MMS doses. (**F**) Quantification of independent experiments as the one in (E). (**G**) RT-qPCR analysis of MED13 mRNA levels upon treatment with increasing MMS concentrations. (**H**) Immunofluorescence analysis of γH2AX foci in HAP1 WT and MED13 KO cl.10 and cl.17, with or without MMS treatment (125 μM, 72 h) Scale bar: 10 μm. (**I**) Signal coverage calculated as mean number of foci from at least 50 analyzed nuclei per sample, per experiment as the one in (H). (**J**) Immunoblot analysis of p53 phophorylated at Ser15 (P-Ser15) and β-actin protein levels in HAP1 WT and MED13 KO extracts. (**K**) Quantification of three independent experiments as the one in (J). (**L**) Apoptotic cell population analysis in HAP1 WT and MED13 KO cl.10, with or without MMS treatment (125 μM, 72h). All error bars indicate mean ± SEM (*n* ≥ 3). Linear regression analysis of dose response in (B–D). One-way ANOVA in (F), (G); two-way ANOVA in (I), (K), (L); *P ≤ 0.05, **P ≤ 0.01, ***P ≤ 0.001, ****P ≤ 0.0001, ns – not significant.

To explore the potential of MED13 in modulation of alkylation resistance (Figure 1), we next generated two independent HAP1 MED13 KO cell lines (MED13 KO cl.10 and cl.17) using CRISPR–Cas9 editing (Figure 2A). By using colony formation and viability assays, we observed that MED13 KO cells were significantly resistant to MMS, when compared to WT cells (Figure 2B and Supplementary Figure S2A), thus confirming the results of CRISPR–Cas9 screen (Figure 1). Similar to MMS, HAP1 MED13 KO cells were also resistant to other alkylating agents TMZ and BCNU (Figure 2C and D, and Supplementary Figure S2B and C). To examine the importance of MED13 in modulation of alkylation response beyond HAP1 cells, we next inactivated *MED13* by CRISPR–Cas9 in glioblastoma G144 cells and generated two independent MED13 KO cell lines (MED13 KO cl.5 and cl.12) (Supplementary Figure S2D). As in HAP1 cells, lack of MED13 resulted in resistance of G144 cells to alkylation by TMZ and MMS (Supplementary Figure S2E and F). Interestingly, besides to alkylating agents, MED13 KO caused resistance to treatment with oxidizing agent hydrogen peroxide (H_2_O_2_), but not to hydroxyurea in HAP1 cells (Supplementary Figure S2G and H). In conclusion, our findings suggest that loss of MED13 promotes resistance to alkylating and oxidizing agents. To determine how alkylation treatment influences endogenous MED13 levels, mRNA and protein expression were analyzed next in HAP1 WT cells. Immunoblot analysis of surviving cell fraction after alkylation exposure revealed that MED13 protein levels are significantly reduced upon both MMS (Figure 2E and F) and TMZ (Supplementary Figure S2J and K) treatments. Similar to the protein, also MED13 mRNA levels were significantly reduced upon the treatment with higher MMS doses, as determined by RT-qPCR analysis (Figure 2G). To determine the impact of MED13 loss on DNA damage response (DDR), immunofluorescence analysis of phosphorylated histone variant H2AX (γH2AX), as mark of DNA breaks, was performed. The DDR is initiated by the ATM kinase at DNA break sites, which generates γH2AX (44). Lack of MED13 interestingly resulted in significantly less γH2AX foci upon the MMS exposure (Figure 2H and I). As H2AX, DDR regulator p53 becomes phosphorylated upon genotoxic stress at sites including Serine 15 (P-Ser15), which leads to p53 stabilization and activation (45). Immunoblot analysis indicated significant reduction in P-Ser15 p53 upon MMS exposure in MED13 KO cells, when compared to WT cells (Figure 2J and K). Accordingly, MED13 KO cells exhibited lowered apoptosis rate upon the treatment with MMS (Figure 2L), while proliferation was unaffected (Supplementary Figure S2I). In summary, these findings indicate that loss of MED13 promotes resistance to alkylation, characterized by significant reduction in DNA breaks and reduced apoptosis upon exposure to alkylating agents.

### MED13 regulates expression of genes centered around cyclin D1

**Figure 3.**
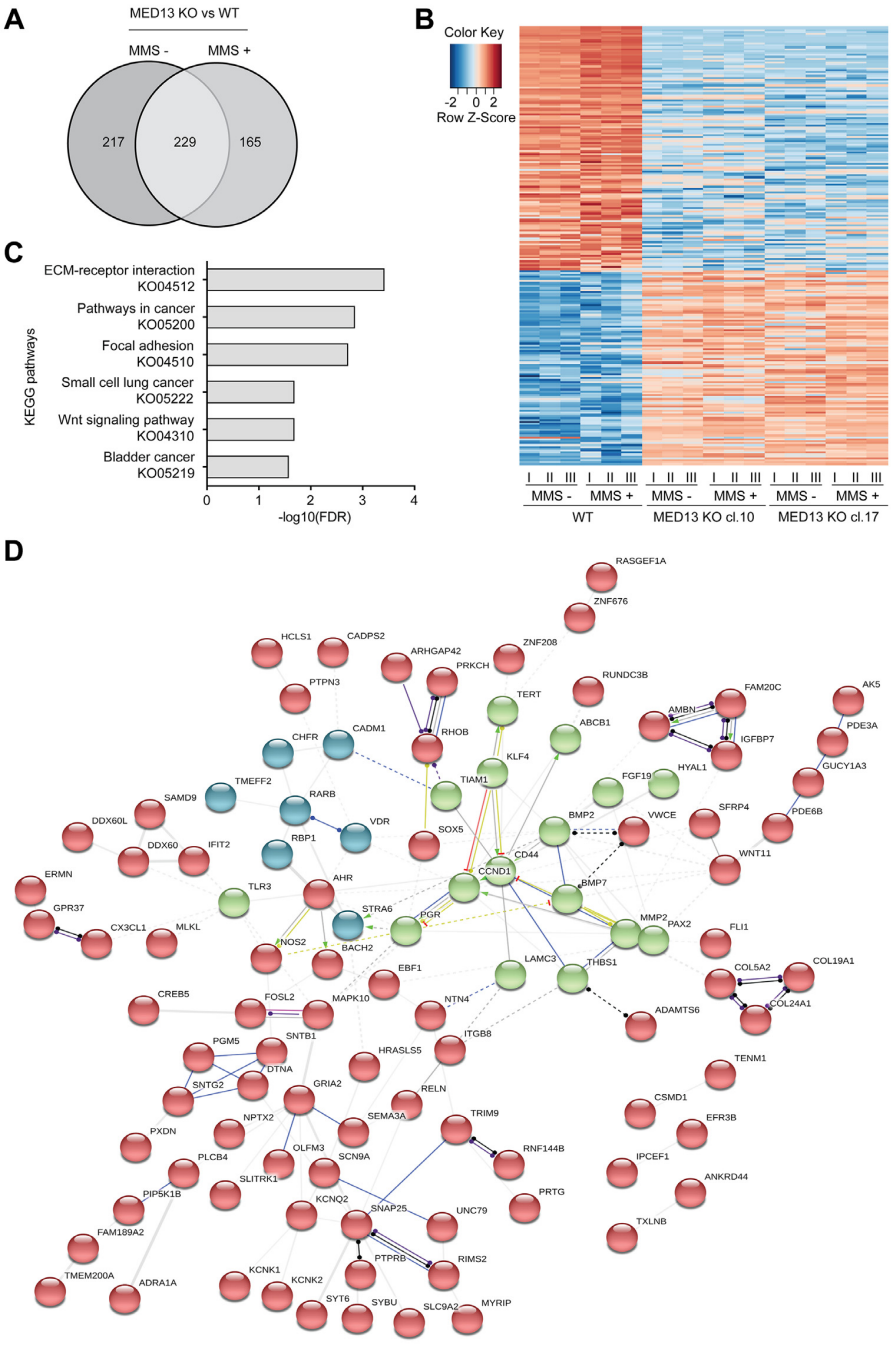
MED13 loss alters expression of genes centered around cyclin D1. (**A**) Venn diagram of differentially expressed genes in HAP1 MED13 knock-out (KO) cl.10 and cl.17, when compared to the wild type (WT) cells, untreated or exposed to methyl methanesulfonate (MMS) (125 μM, 72 h). (**B**) Heat map of common differentially expressed genes in untreated and MMS treated MED13 KO cl. 10 and cl. 17, when compared to the WT HAP1 cells. In each condition three biological replicates (I-III) were analyzed. (**C**) Top six KEGG pathway terms as determined by the Gene Set Enrichment Analysis (GSEA) of 229 common genes regulated by MED13 in untreated and MMS treated cells identified in (A). (**D**) STRING interaction networks functional enrichment analysis of 229 common gene products identified in (A). Neatwork is subdivided in three clusters (depicted in red, green and blue) through k-means clustering, with the members that characterize each cluster presented. Edge thickness is representative of interaction confidence, based on the experimental evidence, data and text mining.

Since MED13 is a subunit of large transcriptional Mediator complex, to investigate a mechanism of MED13 KO resistance to alkylation, we performed RNA sequencing analysis. Transcriptomes of MED13 deficient and proficient cells were compared in the absence and presence of MMS. After bioinformatic processing, at ≥2-fold change and FDR≤0.1, 446 differentially expressed genes (DEGs) were identified in untreated MED13 KO, when compared to WT HAP1 cells (Figure 3A and B). Upon the MMS treatment 394 genes were differentially expressed in MED13 KO in comparison to the WT cells. Of all DEGs, 229 genes were common to both untreated and treated condition, thus representing genes directly regulated by MED13. Since alkylation resistance is a feature of cancer related processes, we next addressed to which pathways MED13 regulated genes belong to. Interestingly, the KEGG pathway analysis revealed that 229 DEGs most significantly segregate to pathways in cancer and signaling processes, including extracellular matrix (ECM) receptor interaction signaling and Wnt pathway (Figure 3C). Next, to determine if products of the 229 DEGs form functional networks STRING enrichment analysis was performed. This analysis showed that numerous products of MED13-regulated genes integrate in a large multi-cluster functional interaction network, in which cell cycle regulator cyclin D1 is one of the central components (Figure 3D). Interestingly, identification of cyclin D1 is in line with the results of the CRISPR–Cas9 screen, which defined cell cycle as one of the most significant BP contributing to the alkylation resistance (Figure 1C). Taken together, our results suggest that loss of MED13 alters expression of multiple genes belonging to cancer-related pathways, and that products of these genes integrated in functional network centered around cyclin D1.

### Cyclin D1 overexpression drives alkylation resistance in cells lacking MED13

**Figure 4.**
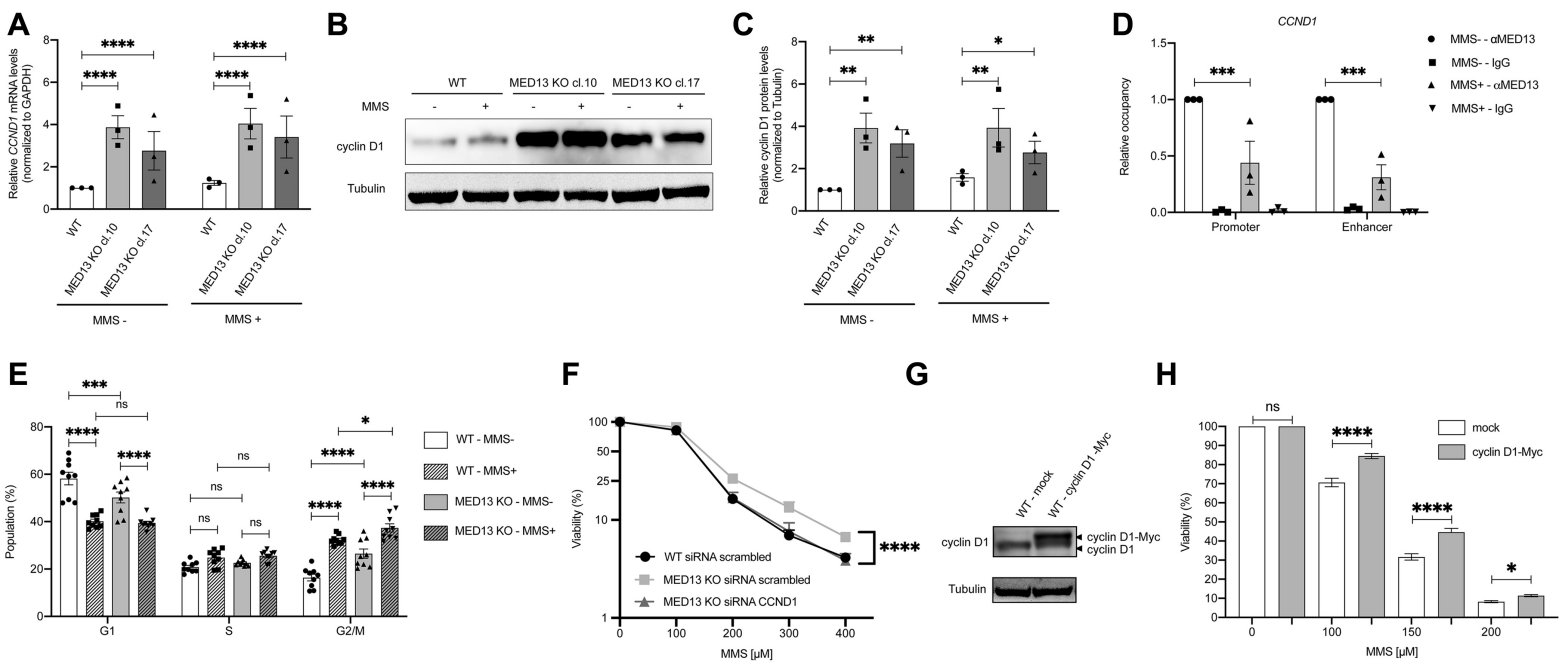
MED13 modulates alkylation response through regulation of cyclin D1 expression. (**A**) RT-qPCR analysis of *CCND1 (cyclin D1*) expression in HAP1 wild type (WT) and two MED13 knock-out (KO) clones (cl.10, cl.17) untreated or exposed to methyl methanesulfonate (MMS) (125 μM, 72 h). (**B**) Immunoblot analysis of cyclin D1 and Tubulin protein levels in WT and MED13 KO HAP1 cells untreated or exposed to MMS (125 μM, 72h). (**C**) Quantification of MED13 protein levels normalized to Tubulin, from experiments as the one in (B). (**D**) ChIP-qPCR analysis of MED13 occupancy at the *CCND1 (cyclin D1*) promoter and enhancer, from cells untreated (NT) or exposed to MMS (125 μM, 72 h). Data is expressed as relative occupancy. (**E**) Cell cycle analysis of HAP1 WT and MED13 KO cells untreated (NT) or exposed to MMS (125 μM, 72 h). (**F**) Viability analysis of HAP1 WT and MED13 KO cells transfected with control scrambled siRNA, or siRNA targeting cyclin D1. (**G**) Immunoblot analysis of cyclin D1 and Tubulin protein levels in cells transfected with control mock or cyclin D1-Myc encoding vector (**H**). Viability of HAP1 WT cells overexpressing cyclin D1-Myc upon 72h treatment with indicated MMS doses. All error bars indicate mean ± SEM (n ≥ 3). Two-way ANOVA statistical testing in (A), (C), (D), (E) and (H); Linear regression analysis of dose response in (F). *P ≤ 0.05, **P ≤ 0.01, ***P ≤ 0.001, ****P ≤ 0.0001, ns - not significant.

To further investigate the impact of MED13 on *CCND1* expression, and consequently cyclin D1 mRNA levels, RT-qPCR analysis was performed in MED13 proficient and deficient cells. In accordance with the RNA sequencing results (Figure 3), cyclin D1 mRNA levels were increased in two independent HAP1 MED13 KO clones cl.10 and cl.17 (Figure 4A), as well as in the two independent G144 MED13 KO clones cl.5 and cl.12 (Supplementary Figure S3A). Loss of MED13 led to *CCND1* overexpression both in the absence and presence of MMS (Figure 4A). In addition to *CCND1*, we confirmed the results of RNA sequencing through analysis of *RELN*, another DEG in MED13 KO cells (Supplementary Figure S3B). Similar to the mRNA levels (Figure 4A), also cyclin D1 protein levels were significantly increased in MED13 KO clones, both untreated and upon MMS exposure (Figure 4B and C). To determine if MED13 has the ability to directly regulate *CCND1* expression, we analyzed next MED13 binding to the regulatory elements. The ChIP-qPCR analysis indicated that MED13 efficiently binds to both the promoter and enhancer of *CCND1* (Figure 4D). Upon MMS treatment MED13 binding was significantly reduced (Figure 4D), further being in line with the globally lowered MED13 protein levels upon the exposure (Figure 2E and F). The specificity of MED13 binding was confirmed by control ChIP-qPCRs in MED13 KO cells (Supplementary Figure S3C). Taken together these results suggest that MED13 has potential to directly regulate *CCND1* expression through binding to its regulatory regions, and consequently that loss of MED13 leads to increased cyclin D1 mRNA and protein levels. Since cyclin D1 overexpression was shown to perturb cell cycle (22), an important component of the alkylation resistance, we next investigated the cell cycle progression in HAP1 WT and MED13 KO cells. In accordance with the cyclin D1 overexpression, the number of HAP1 MED13 KO cells was significantly reduced in the G1 phase of the cell cycle (Figure 4E). MED13 KO cells accumulated in the G2/M phase, which was further promoted by the MMS exposure (Figure 4E). In summary, these findings indicate that cells lacking MED13 overexpress cyclin D1 and are characterized by altered cell cycle progression. While our results suggest that lack of MED13 results in cyclin D1 overexpression, to which extent this influences the alkylation resistance remains unclear. To test this, cyclin D1 was knocked-down in HAP1 MED13 KO cells (Supplementary Figure S4) and the survival upon alkylation exposure analyzed. Importantly, reduced cyclin D1 levels in HAP1 MED13 KO cells resulted in loss of resistance phenotype to MMS treatment (Figure 4F). To test whether cyclin D1 upregulation can induce resistance to MMS, we overexpressed cyclin D1 in HAP1 cells (Figure 4G). As expected, and in line with the knock-down experiment, cyclin D1 overexpression resulted in resistance to MMS, when compared to mock transfection (Figure 4H). Taken together, these results demonstrate that cyclin D1 overexpression in MED13 KO cells is an essential contributor to the alkylation resistance.

### MED13 stabilization, through combinatory CDK8/19 inhibitor treatment, significantly sensitizes cancer cells to alkylation

**Figure 5.**
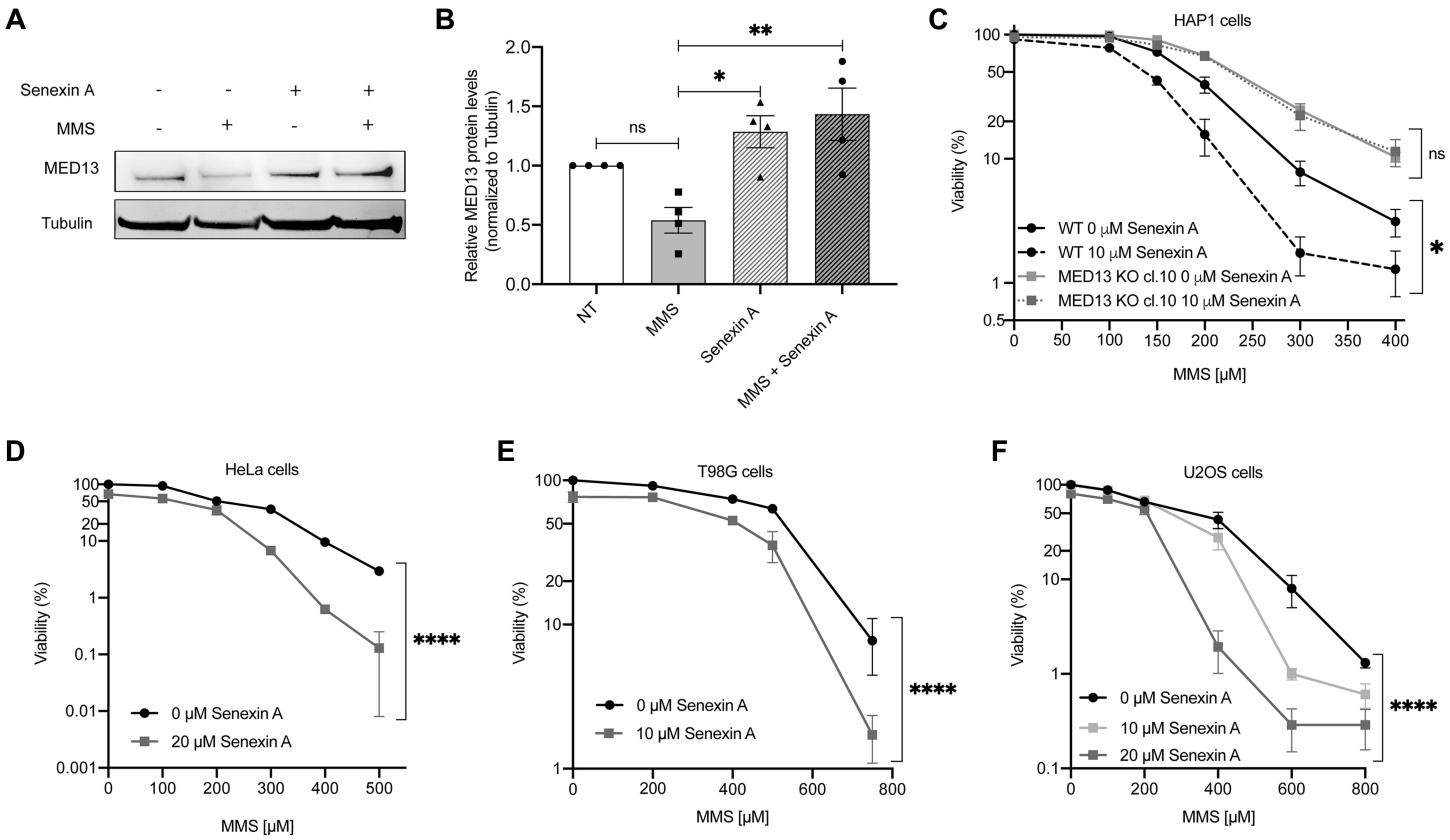
Treatment with CDK8/19 inhibitor Senexin A stabilizes MED13 and sensitizes cancer cells to the alkylation. (**A**) Immunoblot analysis of MED13 and Tubulin protein levels in HAP1 cells untreated (NT) or treated with 150 μM methyl methanesulfonate (MMS), CDK8/19 inhibitor Senexin A (10 μM), or MMS (150 μM) and Senexin A (10 μM) in combination. (**B**) Quantification of four independent experiments as the one in (A). (**C–F**) Viability of HAP1 WT and MED13 KO (C), HeLa (D), T98G (E) and U2OS (F) cells upon treatment with DMSO and Senexin A, in combination with indicated MMS amounts. All error bars indicate mean ± SEM (*n* ≥ 3). One-way ANOVA statistical testing in (B); Linear regression analysis of the dose response in (C–F); *P ≤ 0.05, **P ≤ 0.01, ***P ≤ 0.001, ****P ≤ 0.0001, ns – not significant.

To elucidate the therapeutic potential of MED13-mediated alkylation response, we next tested if MED13 stabilization could sensitize cells to alkylating agents. MED13 phosphorylation at Ser749, mediated by CDK8 (46), was previously shown to be a prerequisite for its proteasomal degradation (47). To stabilize MED13 we thus pre-treated HAP1 cells with CDK8/19 inhibitor Senexin A. The efficiency of Senexin A was confirmed by immunoblot analysis of STAT1 phosphorylation at Ser727 (P-Ser727), a known CDK8/19 target site, which was as expected reduced upon the inhibitor treatment (Supplementary Figure S5A). Importantly, the immunoblot analysis showed that treatment with Senexin A significantly stabilizes MED13 protein levels, and that this effect is maintained upon the combinatory treatment with MMS in HAP1 cells (Figure 5A and B), as well as HeLa adenocarcinoma and T98G glioblastoma cells (Supplementary Figure S5B–D). Moreover, pretreatment with Senexin A significantly reduced viability of both HAP1 and HeLa cells, causing hypersensitivity to alkylation with MMS (Figure 5C and D) and TMZ (Supplementary Figure S5E and F). Importantly, the efficiency of combinatory treatment is directly dependent on MED13 status, since the response of MED13 KO cells to alkylation remains unchanged, irrespective of Senexin A presence (Figure 5C). This suggests that the Senexin A specifically targets MED13 mediated response. Similar to HAP1 and HeLa cells, also glioblastoma T98G and bone osteosarcoma U2OS cells were hypersensitive to the combinatory treatment with Senexin A and MMS (Figure 5E and F). In conclusion, these findings indicate that MED13 stabilization, through CDK8/19 inhibition with Senexin A, significantly sensitizes cancer cells to treatment with alkylating agents.

## DISCUSSION

Alkylating agents belong to the oldest group of chemotherapeutics frequently used to treat cancer. Despite their use in clinics, cancer cells often develop resistance to alkylating drugs (5,6). To improve current treatment strategies, it is essential to broaden knowledge about the factors that drive alkylation resistance. Here, by using CRISPR–Cas9 based genetic screen we identified novel candidate genes, which when inactivated promote resistance to alkylation by MMS (Figure 1). The majority of the most significant candidates belong to the transcription regulation and the cell cycle processes. This finding is in line with the genomic phenotyping performed in yeast, which suggested transcription and cell cycle processes as important modulators of the alkylation resistance (7,8). The top three candidate genes belonging to transcription regulation, include *TNIP2* (fetal liver LKB1-interacting protein), *LMNA* (Lamin A) and *MED13* (Figure 1C-E and Supplementary Table S3). In contrast to *TNIP2* and *LMNA*, which had previously been associated with regulation of signaling and cellular responses to alkylation (48,49), *MED13* had not been linked to alkylation resistance. Our results suggest that loss of MED13 promotes survival upon treatment with different alkylating agents, as well as the oxidizing agent H_2_O_2_ (Figure 2 and Supplementary Figure S2). By analyzing the expression levels in the fraction of surviving cells, we further observe that MED13 levels are reduced upon the MMS exposure (Figure 2E–G). Work in *S. cerevisiae* similarly indicated that MED13 was degraded upon the oxidative stress (50). Since loss of MED13 promotes resistance to DNA damaging agents, we next analyzed DDR in MED13 KO cells. Interestingly, upon MMS treatment both γH2AX and P-Ser15 p53 levels were lower in MED13 KO cells, accompanied by reduced apoptosis (Figure 2). This suggests that lack of MED13 could prevent MMS induced DNA damage, as well as promote DNA repair. Notably, previous work in mouse embryonic fibroblasts (MEFs) demonstrated that lack of another Mediator subunit MED23 similarly results in reduced γH2AX levels, followed by enhanced DNA repair capacity upon UV-induced damage (51). On the mechanistic level, our findings indicate that loss of MED13 alters gene expression and leads to cyclin D1 overexpression (Figure 4 and Supplementary Figure S3A). Cyclin D1 overexpression characterizes numerous human cancers; nearly 90% of mantle cell lymphomas and 50% of breast cancers exhibit cyclin D1 overexpression (52). Fruther, cyclin D1 overexpression, as the one observed in alkylation resistant MED13 KO cells, was shown to be associated with chemo- or radiation- therapy resistance in cancers from patients (53,54). Similar to MED13, downregulation of other Mediator complex subunits MED23, MED1 and MED21 was shown to stimulate *CCND1* (*cyclin D1*) expression (55,56). MED13 can further bind to the promoter and enhancer regions of *CCND1*, thus having the ability to directly regulate its expression (Figure 4D). By analyzing the extent to which cyclin D1 overexpression influences cell cycle progression, we observed reduced number of MED13 KO cells in the G1 phase and increase in G2/M (Figure 4E). Both shortened G1 and accumulation in G2/M phase were shown to result from cyclin D1 overexpression (23,57). Importantly, the elevated cyclin D1 level directly contributes to the alkylation resistance phenotype, since knock-down of cyclin D1 markedly sensitized the MED13 KO cells (Figure 4F), while overexpression of cyclin D1 (Figure 4G-H) induced resistance to the MMS treatment. Consistent with this, it was shown that down-regulation of cyclin D1 expression restores chemo- and radiation- sensitivity in the cancer cells (53,58,59). Based on earlier work, cyclin D1 overexpression could contribute to the alkylation resistance in MED13 KO cells through several different pathways. Cyclin D1 overexpression could potentially promote survival through the positive impact on DNA repair (24,25). Further, increased cyclin D1 levels were suggested to antagonize checkpoint-induced cell cycle arrest upon DNA damage, thus permitting cell division and contributing to the drug resistance (18). Recent work reported that cyclin D1 overexpression results in global transcriptional modulation (60), which could impact the coordination of cell cycle transcription. Taken together, further studies are needed to identify the downstream effectors of cyclin D1 overexpression in MED13 KO cells.

Since downregulation of MED13 is essential to promote survival upon alkylation exposure (Figure 2 and Supplementary Figure S2), we hypothesized that MED13 stabilization can lead to increased sensitivity and cell death. MED13 was previously shown to be targeted for proteasomal degradation in a CDK8-dependent manner, through phosphorylation at Ser749 (46), and subsequent ubiquitination by F-box/WD repeat-containing protein 7 (Fbw7) (47). To stabilize MED13 we thus designed an approach that relies on CDK8 inhibition. Senexin A is a potent and relatively selective inhibitor of CDK8, and its paralogue CDK19 (61). As shown in Figure 5 and Supplementary Figure S5, the pretreatment with Senexin A prior to the MMS treatment stabilizes MED13, and significantly sensitizes HAP1, as well as adenocarcinoma HeLa, glioblastoma T98G, and osteosarcoma U2OS cells to alkylation treatment. These findings strongly support our idea that combinatory treatment composed of CDK8 inhibitor and alkylating agents can have synergistic effects and promote killing of the cancer cells. To date, several CDK8/19 inhibitors have been developed with varying specificity and potency. Inhibitors such as Senexin A, Senexin B, Cortistatin A, SEL120-34A are currently in preclinical testing (62). Several of these inhibitors were recently shown to block transcription of proto-oncogenes like *β-catenin* in human colon cancer HCT116 cells (63,64). Similarly, Senexin B caused growth inhibition of the breast cancer MCF7 cell line (65). While CDK8/19 inhibition has promising perspectives for the cancer therapy, *in vivo* tests with several currently available inhibitors reported severe adverse effects in animals (66). In addition, it is challenging to find an optimal therapeutic window for several of the CDK8/19 inhibitors (66). Thus, there is a clear need for designing and testing of new molecules, which could be less toxic and better tolerated.

Taken together, our findings identified MED13 as a novel modulator of alkylation response, a downregulation of which promotes resistance to DNA damaging agents. Moreover, we provide mechanistic insights to the MED13 mediated alkylation response, by demonstrating link between MED13 status and cyclin D1 expression relevant for the cell survival. Finally, we propose an innovative strategy for potential combinatory treatment with CDK8/19 inhibitors that stabilize MED13, and significantly potentiate cancer cell killing by alkylating drug. The full potential of CDK8 inhibition in combination with alkylating chemotherapeutics remains to be explored.

## DATA AVAILABILITY

The RNA sequencing data reported in this paper are available in GEO under accession GSE147366. Data associated with FACS analysis is available in FlowRepository under accession FR-FCM-Z32R and FR-FCM-Z32T.

## Supplementary Material

gkaa1289_Supplemental_File
