# Supplementary material for: Loss of Mediator complex subunit 13 (MED13) promotes resistance to alkylation through cyclin D1 upregulation
Source: Nucleic Acids Res. 2021 Jan 14;49(3):1470–84. doi: 10.1093/nar/gkaa1289 (PMC7897519; doi:10.1093/nar/gkaa1289)
Supplement: gkaa1289_Supplemental_File [file gkaa1289_supplemental_file.pdf]

Roliński, M. et al.

SUPPLEMENTARY INFORMATION

Supplementary Figure Legends

Supplementary Figure 1. Mediator complex subunits in CRISPR-Cas9 screen for identification of alkylation response regulators. (**A-B**) Scatterplot representation of genes encoding Mediator complex subunits at 3 (A) and 7 (B) days of 125 μM methyl methanesulfonate (MMS) treatment.

Supplementary Figure 2. Role of MED13 in response to different damaging factors. (**A-C**) Viability of HAP1 wild type (WT) and MED13 knock-out (KO) clones after 72h treatment with indicated amounts of methyl methanesulfonate (MMS) (A), temozolomide (TMZ) (B) and 1,3-bis[2-Chloroethyl]-1-nitrosourea (BCNU) (C). (**D**) Immunoblot analysis of MED13 and Tubulin protein levels in G144 WT and MED13 KO clones (cl.5 and cl.12). (**E-F**) Viability of G144 WT and MED13 KO (cl.5 and cl.12) cells upon 72h treatment with indicated amounts of MMS (E) or TMZ (F). (**G-H**) Viability of HAP1 WT and MED13 KO cl.10 and cl.17 upon 72h exposure to H_2O_2 (G) and hydroxyurea (HU) (H). (**I**). Comparison of HAP1 WT and MED13 KO proliferation. (**J**) Immunoblot analysis of MED13 and Tubulin protein levels in HAP1 cells upon 72h exposure to different doses of TMZ. (**K**) Quantification of independent experiments as the one in (J). All error bars indicate mean ± SEM ($n \geq 3$). Linear regression analysis of dose response in (A-C) and (E-G). Two-way ANOVA in (H-I), one-way ANOVA in and (K); *$p \leq 0.05$, **$p \leq 0.01$, ***$p \leq 0.001$, ****$p \leq 0.0001$, ns - not significant.

Supplementary Figure 3. Impact of MED13 on *CCND1* and *RELN* expression. (**A**) RT-qPCR analysis of *CCND1* mRNA levels in G144 wild type (WT) and MED13 knock-out (KO) clones (cl.5 and cl.12). (**B**) RT-qPCR analysis of *RELN* mRNA levels in HAP1 WT and two MED13 KO clones (cl.10, cl.17) treated and non-treated with 125 μM methyl methanesulfonate (MMS) for 72h. (**C**) ChIP-qPCR analysis of MED13 occupancy at the *CCND1 (cyclin D1*) promoter in HAP1 WT and MED13 KO cells. Data is expressed as relative occupancy. Error bars indicate mean ± SEM ($n = 3$). Statistic was calculated using one-way ANOVA in (A) and two-way ANOVA with Tukey multiple comparison test in (B); *$p \leq 0.05$, ns - not significant.

Supplementary Figure 4. Evaluation of siRNA cyclin D1 knock-down efficiency. Immunoblot analysis of cyclin D1 and Tubulin protein levels upon siRNA mediated knock-down of CCND1, 0, 24, 48 and 72 h after siRNA transfection in HAP1 MED13 knock-out (KO) cells.

Supplementary Figure 5. Senexin A sensitizes cells to alkylation treatment. (**A**) Immunoblot analysis of STAT1 Serine 727 phosphorylation (P-S727) and Tubulin levels upon treatment with DMSO control, methyl methanesulfonate (MMS) (150 μM), CDK8/19 inhibitor Senexin A (10 μM), or MMS (150

μM) and Senexin A (10 μM) in combination. STAT1 is phosphorylated by CDK8 at serine 727 and serves as a functional control for Senexin A treatment effectivity. (**B**) Immunoblot analysis of MED13 and Tubulin protein levels in HeLa cells treated for 72h with DMSO control, 200 μM methyl methanesulfonate (MMS), CDK8/19 inhibitor Senexin A (10 μM), or MMS (200 μM) and Senexin A (10 μM) in combination. **(C**) Quantification of independent experiments as the one in (B). (**D**) Immunoblot analysis of MED13 and Tubulin protein levels in T98G cells treated for 72h with with DMSO control, MMS (400 μM), Senexin A (10 μM), or MMS (400 μM) and Senexin A (10 μM) combined. (**E-F**) Viability of HAP1 (D) and HeLa (E) cells upon treatment with indicated amounts of temozolomide (TMZ) in the absence and presence of Senexin A for 72h. All error bars indicate mean $\pm$ SEM ($n \geq 3$). Linear regression analysis of dose response in (E); Two-way ANOVA statistical testing in (F); *$p \leq 0.05$, **$p \leq 0.01$, ***$p \leq 0.001$, ****$p \leq 0.0001$, ns - not significant.

Supplementary Figure 1.

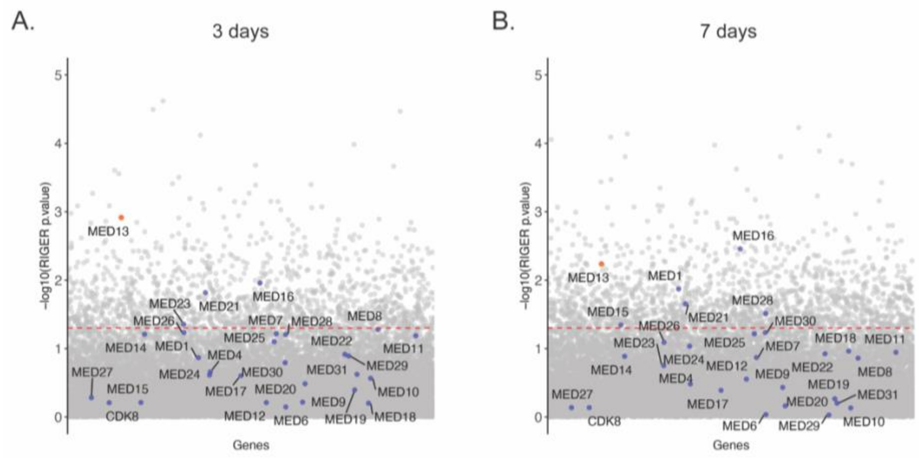

Supplementary Figure 2.

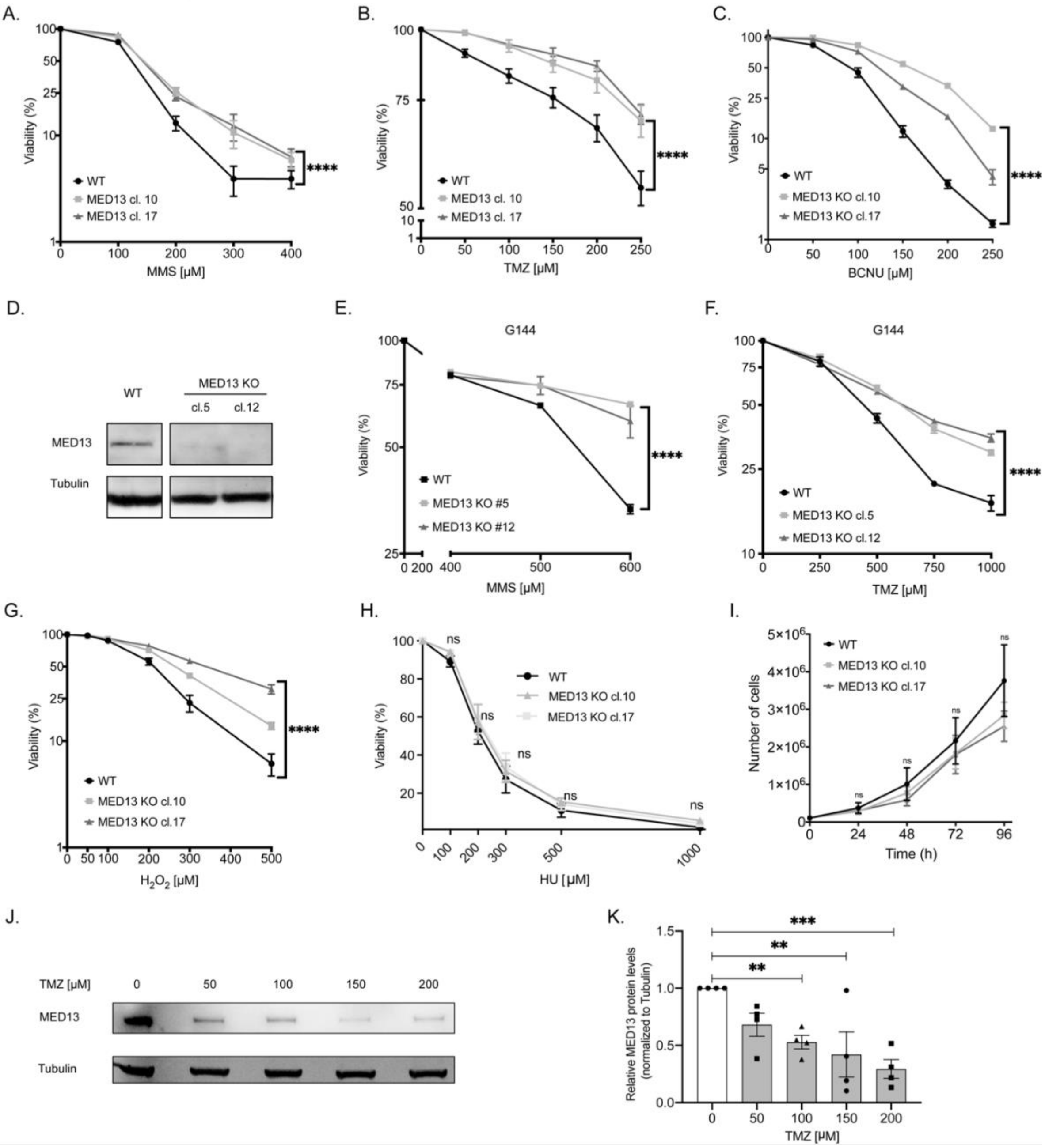

Supplementary Figure 3.

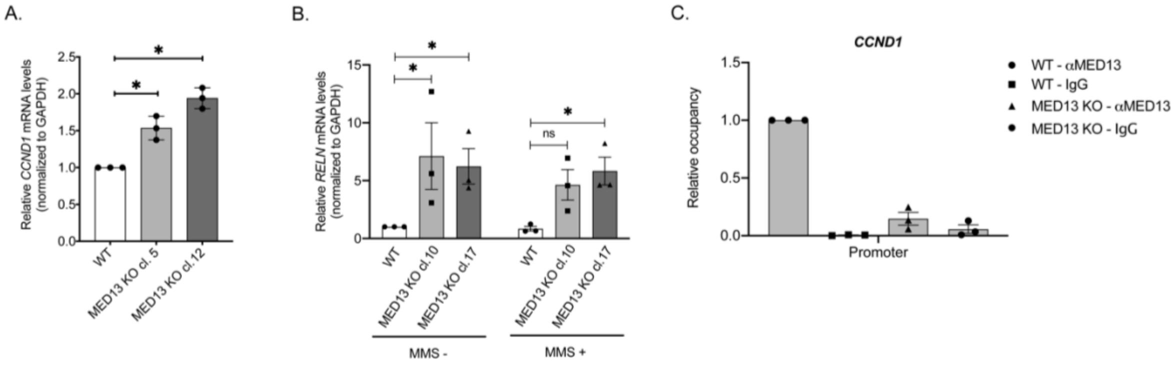

Supplementary Figure 4.

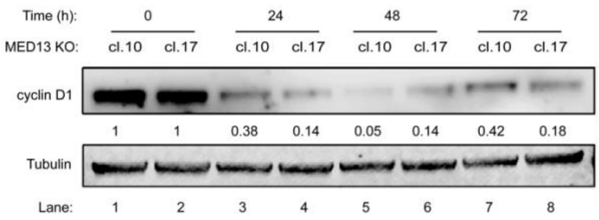

Supplementary Figure 5.

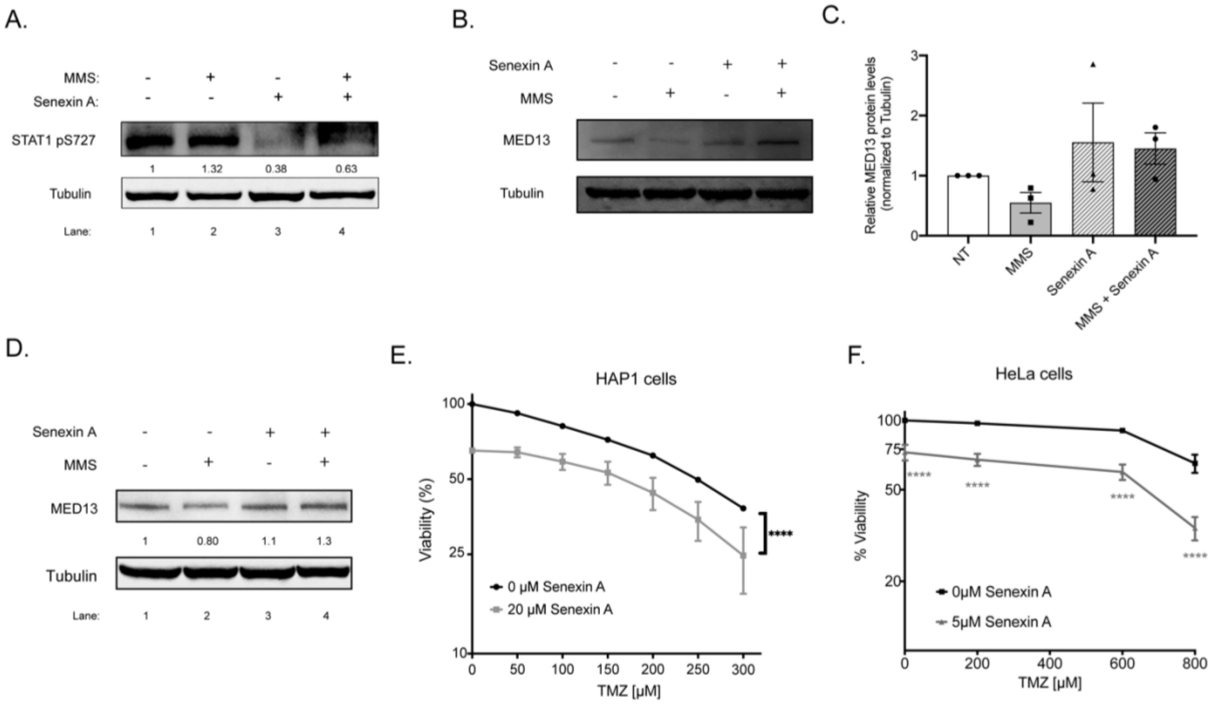

Supplementary Tables

Supplementary Table 1. Oligonucleotides used in the study. Sequences of all primers used, together with the respective applications.

Name	Type	Application	Sequence 5' to 3'
**251_F1-Seq5N8**	DNA	1ST PCR screen	CTTTCCCTACACGACGCTCTTCCGATCTNNNNNNNNGTGGAA AGGACGAAACACCG
**260_R1-Seq7N8**	DNA	1ST PCR screen	GGAGTTCAGACGTGTGCTCTTCCGATCTNNNNNNNNCGACT CGGTGCCACTTTTTC
**R2-TSD701**	DNA	2ND PCR screen	CAAGCAGAAGACGGCATACGAGATCGAGTAATGTGACTGGA GTTCAGACGTGTGCTCTTCCGATC
**R2-TSD702**	DNA	2ND PCR screen	CAAGCAGAAGACGGCATACGAGATTCTCCGGAGTGACTGGA GTTCAGACGTGTGCTCTTCCGATC
**R2-TSD703**	DNA	2ND PCR screen	CAAGCAGAAGACGGCATACGAGATAATGAGCGGTGACTGGA GTTCAGACGTGTGCTCTTCCGATC
**R2-TSD704**	DNA	2ND PCR screen	CAAGCAGAAGACGGCATACGAGATGGAATCTCGTGACTGGA GTTCAGACGTGTGCTCTTCCGATC
**R2-TSD705**	DNA	2ND PCR screen	CAAGCAGAAGACGGCATACGAGATTTCTGAATGTGACTGGA GTTCAGACGTGTGCTCTTCCGATC
**R2-TSD706**	DNA	2ND PCR screen	CAAGCAGAAGACGGCATACGAGATACGAATTCGTGACTGGA GTTCAGACGTGTGCTCTTCCGATC
**R2-TSD707**	DNA	2ND PCR screen	CAAGCAGAAGACGGCATACGAGATAGCTTCAGGTGACTGGA GTTCAGACGTGTGCTCTTCCGATC
**R2-TSD708**	DNA	2ND PCR screen	CAAGCAGAAGACGGCATACGAGATGCGCATTAGTGACTGGA GTTCAGACGTGTGCTCTTCCGATC
**R2-TSD 710**	DNA	2ND PCR screen	CAAGCAGAAGACGGCATACGAGATTTCGCGGAGTGACTGGA GTTCAGACGTGTGCTCTTCCGATC
**R2-TSD 711**	DNA	2ND PCR screen	CAAGCAGAAGACGGCATACGAGATGCGCGAGAGTGACTGGA GTTCAGACGTGTGCTCTTCCGATC
**R2-TSD 712**	DNA	2ND PCR screen	CAAGCAGAAGACGGCATACGAGATCTATCGCTGTGACTGGA GTTCAGACGTGTGCTCTTCCGATC
**R2-TSD 713**	DNA	2ND PCR screen	CAAGCAGAAGACGGCATACGAGATGTCGTGATGTGACTGGA GTTCAGACGTGTGCTCTTCCGATC
**MED13_ TOP_ #1**	DNA	MED13 KO generation	CACCGTGCCTCCTTCGTGCCGAACG
**MED13_BTM_#1**	DNA	MED13 KO generation	AAACCGTTCGGCACGAAGGAGGCAC
**MED13_FW**	DNA	Sanger sequencing,	GTAATGGCGGATGGTGGGTT
**MED13_RV**	DNA	Sanger sequencing,	GCCAACACTCCCCTAAACAGA
**MED13 FW qPCR**	DNA	RT-qPCR	GTGAACACTTGTCCTGCTCC
**MED13 RV qPCR**	DNA	RT-qPCR	TGGGCATAAGATAACTTGAAATGGG
**RELN_FW**	DNA	RT-qPCR	CAACCCCACCTACTACGTTCC
**RELN_RV**	DNA	RT-qPCR	TCACCAGCAAGCCGTCAAAAA
**CCND1_FW**	DNA	RT-qPCR	TGAGGCGGTAGTAGGACAGG
**CCND1_RV**	DNA	RT-qPCR	GACCTTCGTTGCCCTCTGT
**GAPDH_Fw**	DNA	RT-qPCR	GAGTCAACGGATTTGGTCGT
**GAPDH_Rv**	DNA	RT-qPCR	TTGATTTTGGAGGGATCTCG
**siRNA scrambled**	RNA	siRNA KD	s: AAGUCAAUAUGCGACUGAUGG[dT][dT] as: CCAUCAGUCGCAUAUUGACUU[dT][dT]
**siRNA_CCND1**	RNA	siRNA KD	s: GCAUGUUCGUGGCCUCUAA[dT][dT] as: UUAGAGGCCACGAACAUGC[dT][dT]
**CCND1_FV_ promotor**	DNA	ChIP -qPCR	CGCATGCTAAGCTGAAATCCC
**CCND1_RV_ promotor**	DNA	ChIP-qPCR	TTTCATTCCGGCGCACAG

**CCND1_FV_ enhancer**	DNA	ChIP -qPCR	ATTCATGGCTGCAGGACCGA
**CCND1_RV_ enhancer**	DNA	ChIP-qPCR	TTCATGAATGGCCCCGCTTC

Supplementary Table 2. **Representation of all significant genes assigned to specific biological processes.** The exact number of genes identified by CRISPR-Cas9 screen in top 5 biological process (BP) categories (from GSEA analysis), and the % of these genes within each BP category. Associated with Figure 1C.

GO: Biological process	# of genes/genes in GO category	% genes in GO category
**Organonitrogen compound biosynthetic process**	55/1839	2,99
**Cell cycle process**	45/1383	3,25
**Small molecule metabolic process**	51/1688	3,02
**Cell cycle**	53/1847	2,87
**Positive regulation of gene expression**	54/1974	2,74

Supplementary Table 3. List of top candidate genes and their biological processes. List of 14 most significant genes ($p < 0.01$) and their biological processes, at both 3- and 7-day time point after MMS treatment. Determined by GSEA gene ontology analysis. Associated with Figure 1D and E.

GO: Biological process	Genes
**Organonitrogen compound biosynthetic process**	*RAB3GAP1, DHPS, EIF4G2*
**Cell cycle process**	*RRM2, PCGF2,EIF4G2, MLH1, LMNA*
**Small molecule metabolic process**	*TTR,RRM2,GNPDA2, AHCY*
**Cell cycle**	*RRM2, PCGF2,EIF4G2, MLH1, LMNA*
**Positive regulation of gene expression**	***MED13***, *RAB3GAP1, TNIP2, LMNA, BCAS3, BATF*
